# Reevaluating the relevance of ^18^F-FDG PET findings for diagnosis of neurosarcoidosis: a case series

**DOI:** 10.1186/s42466-023-00299-9

**Published:** 2024-01-25

**Authors:** Jessy Chen, Giulia Metzger, Christian Furth, Georg Bohner, Volker Siffrin

**Affiliations:** 1 Department of Neurology and Experimental Neurology, Charité Universitätsmedizin Berlin, Augustenburger Platz 1, 13353 Berlin, Germany; 2 Department of Nuclear Medicine, Charité Universitätsmedizin Berlin, corporate member of Freie Universität Berlin and Humboldt Universität zu Berlin, and Berlin Institute of Health, Berlin, Germany; 3 Institute of Neuroradiology, Charité Universitätsmedizin Berlin, Berlin, Germany; 4 Experimental and Clinical Research Center, Charité Universitätsmedizin Berlin and Max Delbrück Center for Molecular Medicine, Berlin, Germany; 5 Berlin Institute of Health at Charité – Universitätsmedizin Berlin, Biomedical Innovation Academy, BIH Charité Clinician Scientist Program, Berlin, Germany

**Keywords:** Neurosarcoidosis, Fluorodeoxyglucose positron emission, Metabolic imaging, Imaging discordance, Diagnostic criteria

## Abstract

**Objective:**

The diagnosis of neurosarcoidosis (NS) remains challenging due to the difficulty to obtain central nervous system (CNS) biopsies. Various diagnostic parameters are considered for the definition of possible, probable and definite NS. Magnetic resonance imaging (MRI) is the imaging gold standard and considered in diagnostic criteria. Fluorodeoxyglucose positron emission (^18^F-FDG PET) is sometimes performed additionally to identify possible systemic biopsy targets. However, at present, its findings are not incorporated into the diagnostic criteria for neurosarcoidosis (NS).

**Methods:**

We conducted a single center retrospective search for the period 2020–2022, for patients with neurological symptoms in a diagnostic context of suspected NS who underwent MRI and additional ^18^F-FDG PET scans to identify potential hypermetabolism in the CNS and biopsy targets.

**Results:**

We identified three cases of NS, where Gadolinium-enhanced MRI scans did not show abnormalities while ^18^F-FDG PET revealed hypermetabolic lesions in areas of the CNS. Additional MRI scans were still inconclusive for structural changes. We diagnosed a “probable” NS in all cases with histopathological confirmation of systemic sarcoidosis which led to an intensified therapy regime.

**Discussion:**

^18^F-FDG PET is an early indicator for metabolic changes. It appears to be a useful add-on to improve accuracy of diagnostic criteria in suspected NS without MRI findings.

## Introduction

Sarcoidosis is an inflammatory disease typically characterized by formation of epithelioid cell granuloma in various organs, with around 25–35% neurological involvement (neurosarcoidosis, NS) [1]. In contrast to lung or lymph node affection, NS has a higher rate of relapse probability and more severe consequences for long-term disability. Therefore, stronger treatment regimens are commonly necessary for controlling neurosarcoidosis [2]. Granulomatous inflammation sites may reside in any central or peripheral nervous structures (CNS, PNS) leading to very heterogeneous clinical manifestations and thus often pose a diagnostic challenge. Diagnostic tools typically include the analyses of blood and cerebrospinal fluid (CSF), magnet resonance imaging (MRI) of the CNS and further electrophysiological tests (neurography, myography). Fluorodeoxyglucose positron emission tomography (^18^F-FDG PET) is an established sensitive method to detect increased cell metabolism. In a review of previous literature, we identified three case reports describing discordances between contrast enhanced MRI and ^18^F-FDG PET, suggesting additional value of ^18^F-FDG PET to diagnose NS [3–5]. However, to date ^18^F-FDG PET findings are currently not taken into account when making a diagnosis for possible, probable or definite NS [6].

This report describes three cases where findings by ^18^F-FDG PET were relevant for the diagnosis of NS, underlining the question if ^18^F-FDG PET findings should be considered as a criterion for NS.

## Case presentation

From 2020–2022 we observed three cases of probable NS, where Gadolinium-enhanced MRI scans did not show any abnormalities while ^18^F-FDG PET revealed hypermetabolic lesions in areas of the CNS. Two patients had a new onset of neurologic symptoms of unknown etiology without other systemic symptoms. The remaining patient was a referral with known systemic sarcoidosis but till then undiagnosed NS. Clinical manifestations were heterogeneous ranging from visual disturbance to sensory spinal cord syndrome or spastic/ataxic gait disorder (Table 1). All these patients presented with a pleocytosis in the CSF. Since MRI was negative or inconclusive ^18^F-FDG PET was performed to (1) identify MRI-negative CNS inflammation and/or (2) screen for the extent of systemic inflammatory sites in order to evaluate best medical treatment options (Table 1).

**Table 1:** Diagnostic results of three patients

Characteristics	Patient 1	Patient 2	Patient 3
Sex	Female	Female	Male
Concomitant diseases	Hypothyreosis, hypertension	Depression	Known systemic sarcoidosis
Neurological symptoms	Visual impairment, headache	Sensory spinal cord syndrom	Spastic atactic gait disorder Bladder dysfunction
Magnetic resonance imaging (MRI)	Unsuspicious, no enhancement	Unsuspicious, no enhancement	Unsuspicious, no enhancement
Positron emission tomography (^18^F-FDG PET)	Spinal cord hypermetabolism (cervical/thoracical) bihilar lymphadenopathy	Spinal cord hypermetabolism (cercival)	Cerebral hypermetabolism (precuneus)
Histopathology	LN epitheloid granuloma	LN epitheloid granuloma	LN epitheloid granuloma (diagnosed before)
Extraneuronal manifestations	Bihilar lymphadenopathy	Bihilar lymphadenopathy	Pulmonary, renal and osseus
Electrophysiology (electromyography/-neurography, evoked potentials)	Visual evoked potential: prolonged latency	Not obtained	Somatosensory evoked potential: prolonged latency
Blood	sIL2R normal autoimmune and infectious test parameters negative	sIL2R elevation, autoimmune and infectious test parameters negative	sIL2R elevation autoimmune and infectious test parameters negative
Cerebral spinal fluid	Pleocytosis, protein elevation, NFL elevation sIL2R normal, no Ig sysnthesis, CD4/CD8 elevated	Pleocytosis, protein elevation, sIL2R elevation, CD4/CD8 normal, no IG synthesis	Pleocytosis, protein elevation, sIL2R elevation, NFL elevation, no Ig synthesis, CD4/CD8 normal
Benefits from ^18^F-FDG PET	Identification of biopsy target leading to diagnosis Initiation of immunodsuppressive treatment targeting at neurosarcoidosis	Initiation of immunosuppressive treatment targeting at neurosarcoidosis	Escalation of immunosuppressive treatment regime targeting at neurosarcoidosis
Treatment; follow up duration	Steroid pulse, methotrexate and prednisolone; 2,5 years	Steroid pulse, methotrexate and prednisolone; 2 years	Steroid pulse, mycophenolate-mofetil and prednisolone; 2 years

*LN* lymph node, *sIL2R* soluble Interleukin 2 receptor, *NFL* neurofilament light chain

**Fig. 1:**
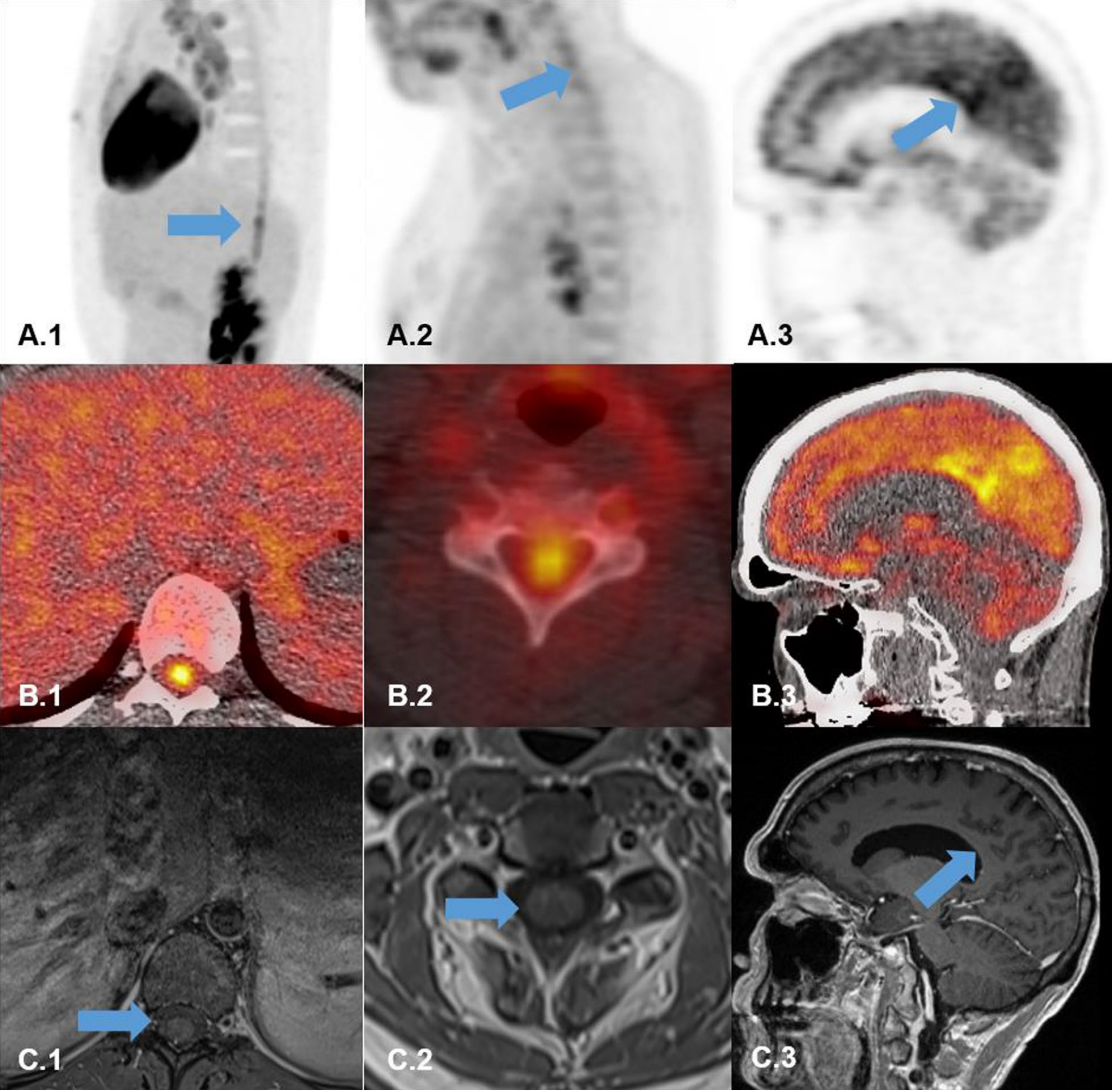
^18^F-FDG PET and MRI scans of three patients. **A.1**–**B.1** ^18^F-FDG PET (A1 sagittal PET Data; B1 transaxial fused PET/CT-Data) showing intense hypermetabolism in the lower thoracical spinal cord (blue arrow). **C.1** Transaxial MRI-scan (T1_TSE_FS) showing no abnormal Gadolinium enhancement in the lumbar spinal cord (blue arrow). **A.2**–**B.2** ^18^F-FDG PET (A2 sagittal PET Data; B2 transaxial fused PET/CT-Data) showing hypermetabolism in the cervical spinal cord (blue arrow). **C.2** Transaxial MRI-scan (T1_TSE_FS) showing no abnormal Gadolinium enhancement in the cervical spinal cord (blue arrow). **A.3**–**B.3** ^18^F-FDG PET (A3 sagittal PET Data; B3 transaxial fused PET/CT-Data) showing bilateral hypermetabolism the precuneus region (blue arrow). **C.3** Transaxial MRI-scan (T1_MPRAGE) showing no abnormal Gadolinium enhancement in precuneus region (blue arrow)

In the CNS, we identified areas of increased tracer uptake in the cervical and thoracic spinal cord in two patients. The other case presented with increased uptake within the precuneus area bilaterally (Fig. 1). Pulmonary and lymph node manifestations of sarcoidosis were already diagnosed during chest X-ray and computed tomography (CT) scan in two cases (patient 2 and 3). Patient 1 was first diagnosed by ^18^F-FDG PET scan with pulmonary lymph node manifestation. All of them had histopathological confirmation of suspected sarcoidosis in a lymph node biopsy. Neural tissue was not obtained due to sensitive regions. In knowledge of ^18^F-FDG PET abnormalities, an additional MRI scan was performed in every patient with non-standard VIBE sequences for spinal cord imaging, but remained inconspicuous. A follow up ^18^F-FDG PET scan was not performed. We diagnosed all three patients with a “probable” NS taking the positive 18F-FDG PET scans as an imaging substitute for MRI results. Patients 1 and 2 were started on immunosuppressive treatment with a steroid pulse resulting in a remission of symptoms in Patient 2 and visual improvement with residual deficits in Patient 1. Both received oral prednisolone in a tapering dose (1 mg/kg bodyweight) with additional azathioprine or methotrexate as a long-term treatment. The treatment regime for patient 3 was changed from methotrexate to mycophenolate-mofetil with additional oral prednisolone tapering dose (1 mg/kg bodyweight). In our outpatient clinic follow up (2–2.5 years depending on the patient case), all three patients remained stable without new disease relapses. Patient 1 is currently treatment-free after two years of treatment and remains stable. sIL2R as a suggestive disease activity marker was normal in a routine control.

## Discussion

We present clinical and imaging data of three patients with “probable” NS. In all patients, we observed discordances between the imaging standard (Gadolinium-enhanced MRI) and metabolic imaging using ^18^F-FDG PET in the CNS. In the end, all presented cases had histopathological lymph node confirmation of sarcoidosis.

The review of literature underlined the relevance of ^18^F-FDG PET findings in the diagnostic pathway with three similar cases reported from 2012 on. Recently, the clinical utility of ^18^F-FDG PET was investigated in 110 patients with suspected NS who underwent ^18^F-FDG PET in a retrospective single-center approach [7]. In total, 27 of 110 patients were diagnosed with possible, probable or definite NS. Remarkably, upon those 19 of 27 (70%) patients with NS had ^18^F-FDG PET avidities suspicious of NS. Also, among those 27 patients, certain ^18^F-FDG PET and MRI discordances were observed. Authors conclude, that ^18^F-FDG PET should be included to the diagnostic evaluation to facilitate a “probable” NS diagnosis if other imaging modalities remain inconclusive. With our clinical experience, we observe a certain group of patients at higher risk for prolonged diagnosis of NS, namely those with (1) exclusive neurological symptoms suggestive of chronic inflammatory disease but inconclusive MRI scans and those with (2) known sarcoidosis, unclear neurological symptoms and inconclusive MRI scans. ^18^F-FDG PET provides a useful supportive diagnostic tool in cases where MRI and CT scans remain inconclusive concerning CNS inflammation. ^18^F-FDG PET is a highly sensitive imaging test for changes of glucose metabolism and may detect inflammatory changes better than standard MRI imaging. Concerning MRI imaging modalities, non-standard contrast enhanced axial VIBE sequences for spinal cord imaging might be a more sensitive way to detect subtle inflammatory leptomeningeal enhancement and should be considered when NS is suspected. However, ^18^F-FDG PET avidities especially in the CNS need to be carefully interpreted because they are not specific to differentiate NS from other e.g. inflammatory conditions. We believe that systemic tracer uptake (e.g. pulmonary) paired with CNS avidity is helpful as a criterion suggestive of NS. The result of isolated positive 18F-FDG PET findings in the CNS pose a diagnostic challenge because the difficulty of obtaining tissue for histopathological analysis remains. Therefore, ^18^F-FDG PET results need to be interpreted in the overall diagnostic context when a chronic-inflammatory condition is suggestive after the canonical diagnostic workflow. However, with limited data available further (prospective) studies are needed to elucidate the possible gain in the accuracy of the diagnosis of NS by ^18^F-FDG PET; thus, a systematic and prospective setting should be emphasized.

## Data Availability

The datasets used and/or analysed during the current study are available from the corresponding author on reasonable request.

## Abbreviations

CNS: Central nervous system
FDG-PET: Fluorodeoxyglucose positron emission tomography
MRI: Magnet resonance imaging
NFL: Neurofilament light chain
NS: Neurosarcoidosis
sIL2R: Soluble interleukin 2 receptor

## References

[1] Bradshaw MJ, Pawate S, Koth LL, Cho TA, Gelfand JM (2021). Neurosarcoidosis. Pathophysiology, Diagnosis, and Treatment.

[2] Voortman M, Drent M, Baughman RP (2019). Management of neurosarcoidosis: A clinical challenge. Current Opinion in Neurology.

[3] Bartels S, Kyavar L, Blumstein N (2013). FDG PET findings leading to diagnosis of neurosarcoidosis. Clinical Neurology and Neurosurgery.

[4] Huang JF, Aksamit AJ, Staff NP (2012). MRI and PET imaging discordance in neurosarcoidosis. Neurology.

[5] Wang Y, Andrews J, Jenkins Colon P, Wundes A (2018). FDG-PET abnormalities leading to the diagnosis of an unusual case of probable neurosarcoidosis. Neurology - Neuroimmunology Neuroinflammation.

[6] Stern BJ, Royal W, Gelfand JM (2018). Definition and consensus diagnostic criteria for neurosarcoidosis: From the neurosarcoidosis consortium consensus group. JAMA Neurology.

[7] Fritz D, van de Beek D, Brouwer MC, Booij J (2020). Whole-body 18F-FDG PET-CT in the diagnosis of neurosarcoidosis. Mayo Clinic Proceedings.

